# Supplementary material for: Theranostic near-infrared-IIb emitting nanoprobes for promoting immunogenic radiotherapy and abscopal effects against cancer metastasis
Source: Nat Commun. 2021 Dec 9;12:7149. doi: 10.1038/s41467-021-27485-0 (PMC8660774; doi:10.1038/s41467-021-27485-0)
Supplement: Supplementary file 1 — Supplementary Information [file 41467_2021_27485_MOESM1_ESM.docx]

Supplementary Information for “Theranostic near-infrared-IIb emitting nanoprobes for promoting immunogenic radiotherapy and abscopal effects against cancer metastasis”

Hao Li^1,4^, Meng Wang^2,4^, Biao Huang^3^, Su-Wen Zhu^1^, Jun-Jie Zhou^1^, De-Run Chen^1^, Ran Cui^3,*^, Mingxi Zhang^2,*^, Zhi-Jun Sun^1,*^

^1^The State Key Laboratory Breeding Base of Basic Science of Stomatology (Hubei-MOST) & Key Laboratory of Oral Biomedicine Ministry of Education, School & Hospital of Stomatology, Wuhan University, 430079 Wuhan, China.

^2^State Key Laboratory of Advanced Technology for Materials Synthesis and Processing, Wuhan University of Technology, 430070 Wuhan, China.

^3^College of Chemistry and Molecular Sciences, Wuhan University, 430072 Wuhan, China.

^4^These authors contributed equally: Hao Li, Meng Wang.

^*^email: cuiran@whu.edu.cn; mxzhang@whut.edu.cn; sunzj@whu.edu.cn


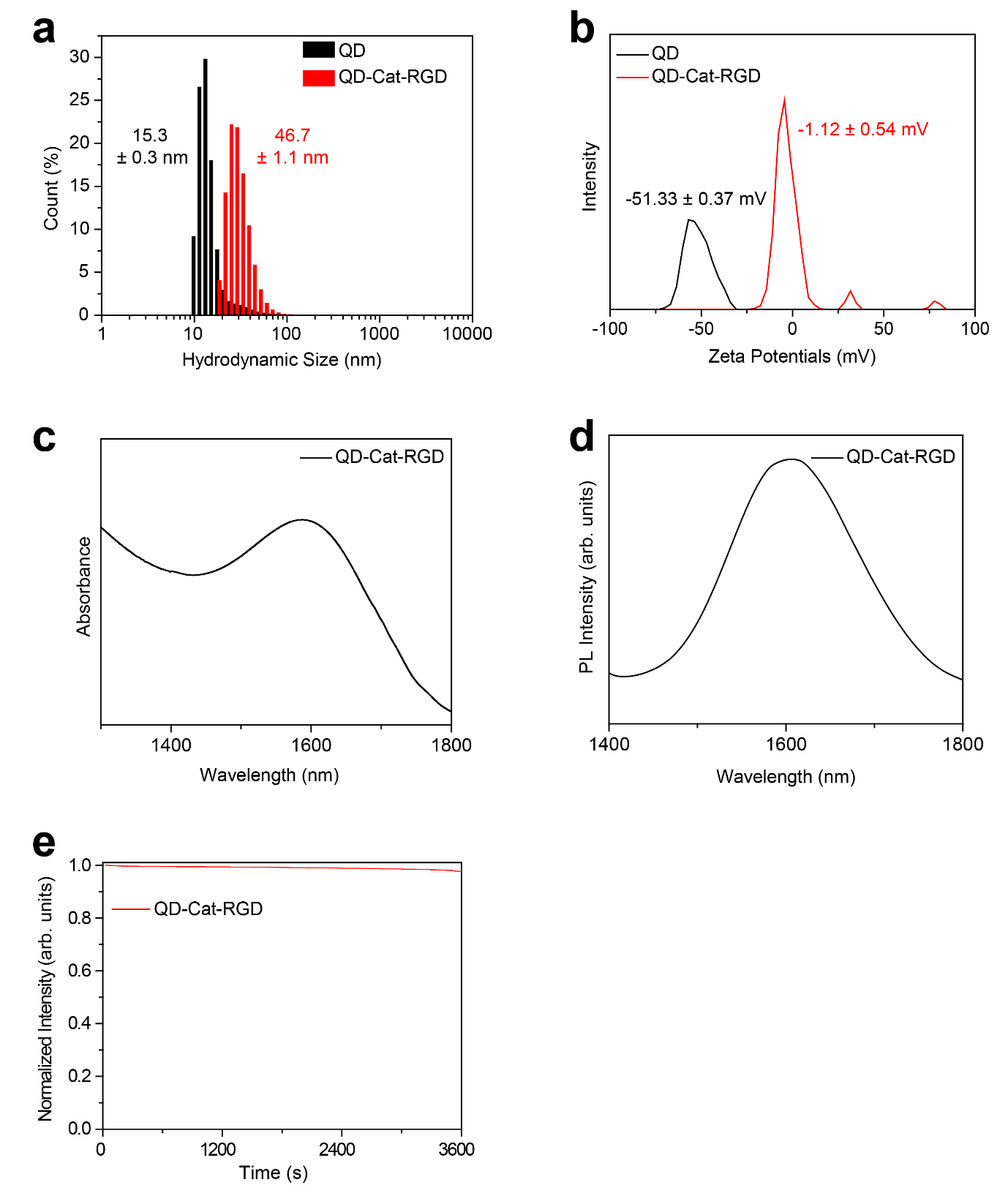


**Supplementary Fig. 1** **a** The hydrodynamic diameters of QD and QD-Cat-RGD quantum dots measured by Dynamic light scattering (DLS). **b** The zeta potential of QD and QD-Cat-RGD. **c** The absorption spectrum of QD-Cat-RGD. **d** The fluorescence emission spectrum of QD-Cat-RGD. **e** The fluorescence intensity of QD-Cat-RGD in fetal bovine serum (FBS) under continuous 808 nm laser exposure (25 mW cm^-2^) for 1 h. arb. units, arbitrary units. Source data are provided as a Source Data file.


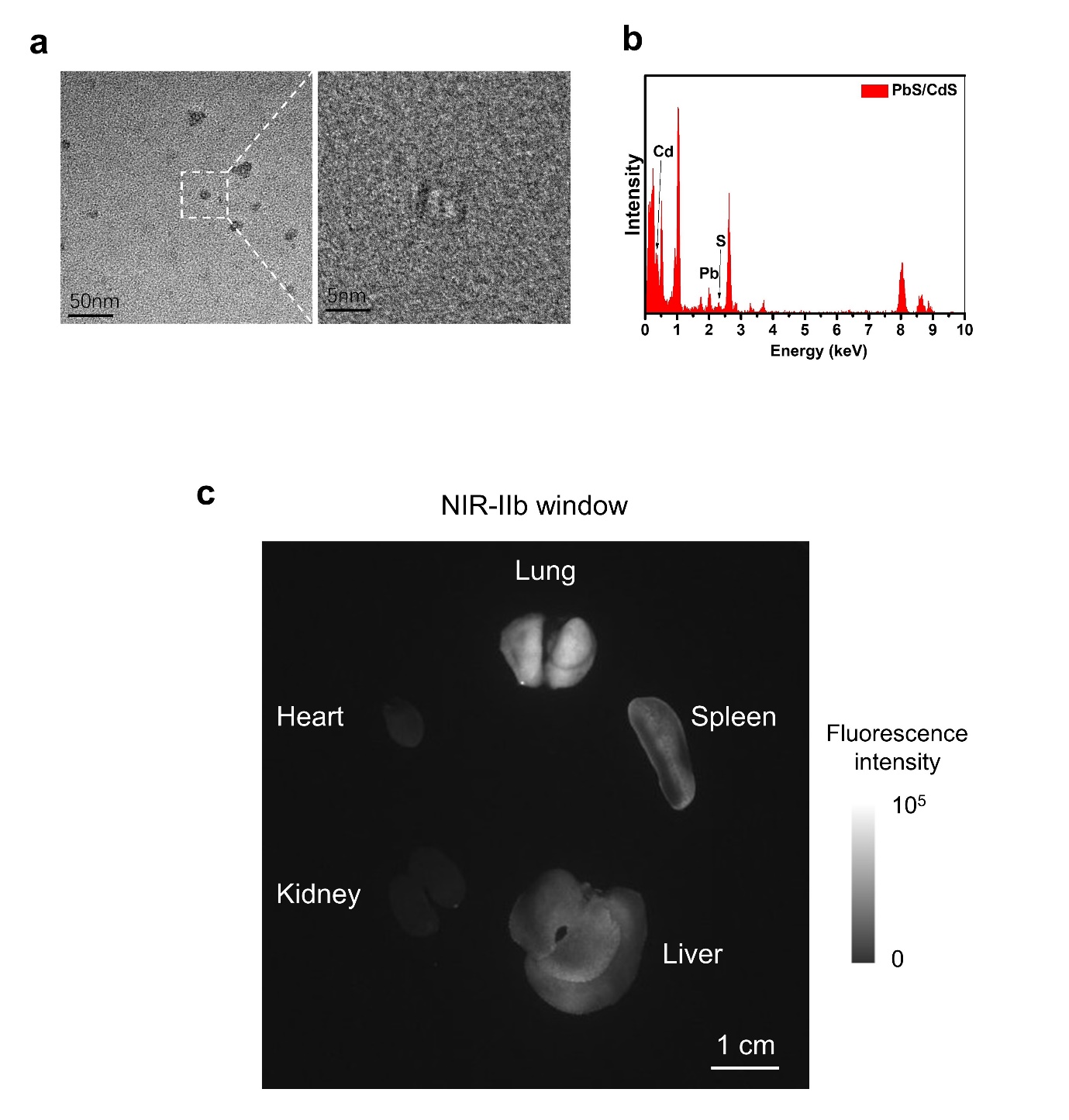


**Supplementary Fig. 2 a** Transmission electron microscopy (TEM) image of QD-Cat-RGD nanoprobes extracted from the feces. **b** Energy-dispersive X-ray spectroscopy spectrum (EDS) of nanoprobes from feces. **c** Fluorescence intensity in the NIR-IIb window of different organs. Scale bar = 1 cm. The excitation power density for NIR-IIb fluorescence imaging was 25 mW cm^-2^ provided by an 808 nm laser. The TEM of the QD-Cat-RGD nanoprobe (**a**) and the fluorescent image of organs in the NIR-IIb window (**c**) were representative of those generated from three independent experiments. Source data are provided as a Source Data file.


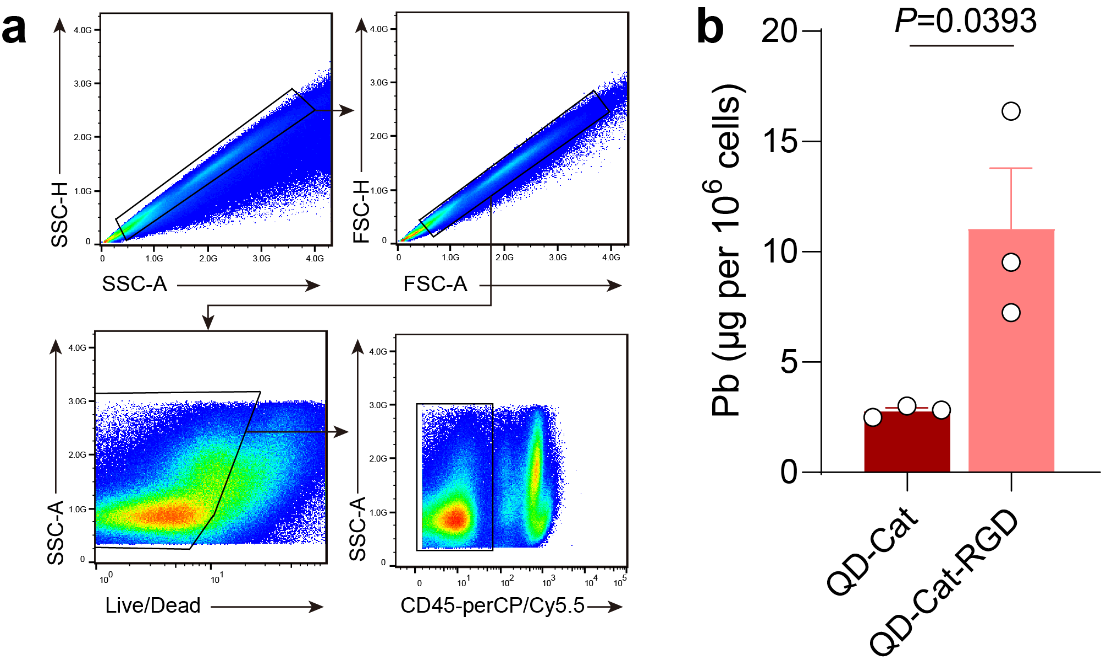


**Supplementary Fig. 3** Tumor tissue harvested from 4T1 tumor-bearing mice which intravenously injected with QD-Cat-RGD (150 μL, 2 mg mL^-1^) or QD-Cat (150 μL, 2 mg mL^-1^) nanoprobes at 4h. Then the live CD45^-^ cells were sorted by fluorescent-activated cell sorting (FACS). **a** The sorting gating strategy of cells. **b** The content of Pb in the live CD45^-^ measured by ICP-OES. All data are shown as the mean ± s.e.m. (n = 3) and n represents the number of independent samples. Statistical significance was calculated via two-tailed Student’s *t* test. Source data are provided as a Source Data file.


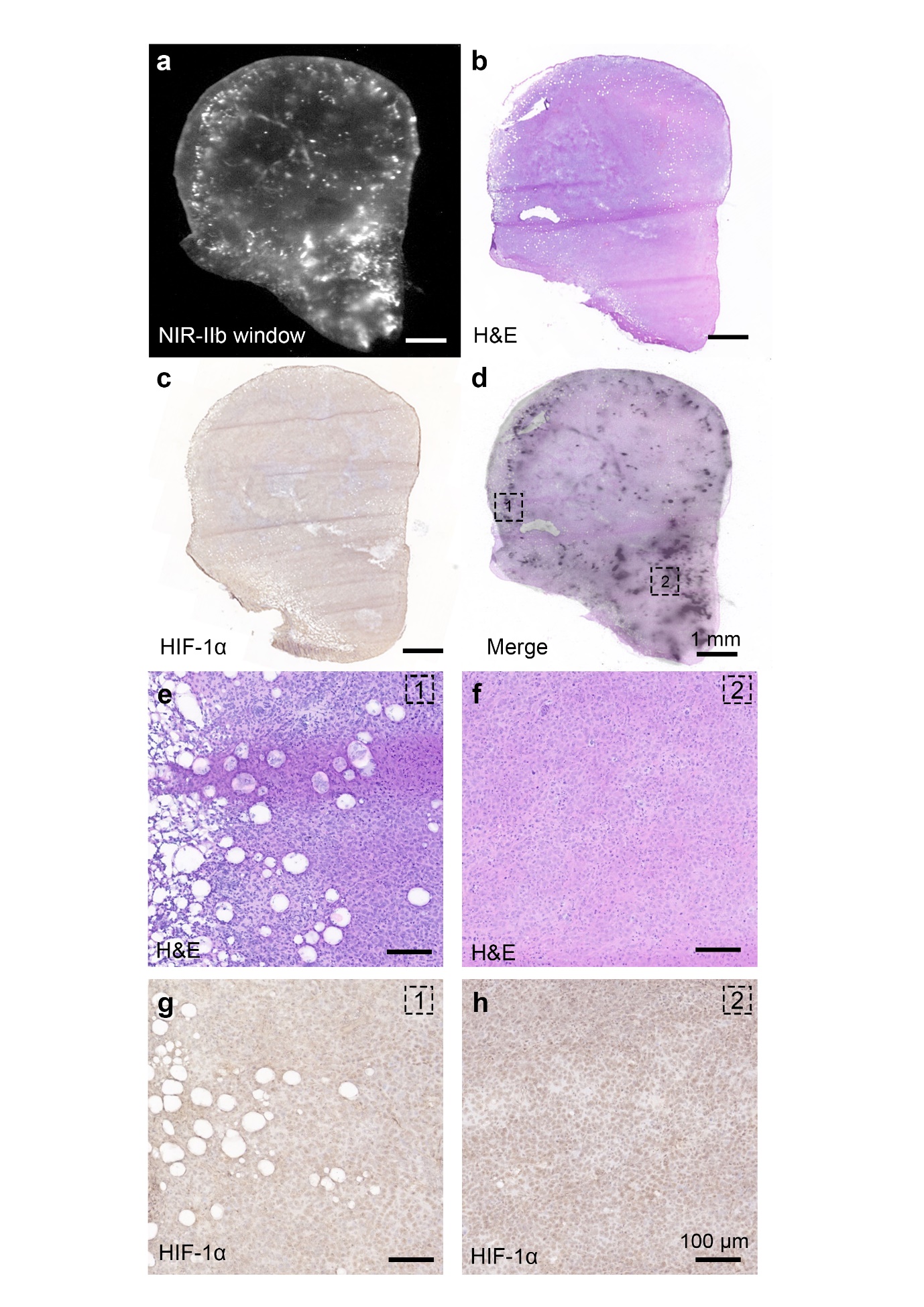


**Supplementary Fig. 4** Tumor tissue harvested from 4T1 tumor-bearing mice which were intravenously injected with QD-Cat-RGD nanoprobes at 4h. **a-d** Serial sections of tumor tissue imaged in the NIR-IIb window, stained with hematoxylin and eosin (H&E) and immunostained with HIF-1α. The excitation power density for NIR-IIb fluorescence imaging was 50 mW cm^-2^ provided by an 808 nm laser. Scale bars = 1 mm. **e, g** H&E staining (**e**) and immunohistochemical staining with HIF-1α (**g**) of the Region 1. Scale bars = 100 μm. **f, h** H&E staining (**f**) and immunostaining with HIF-1α (**h**) of the Region 2. Scale bars = 100 μm. The images of NIR-IIb imaging, H&E staining and immunostaining (**a-h**) were representative of those generated from three mice each group. Source data are provided as a Source Data file.


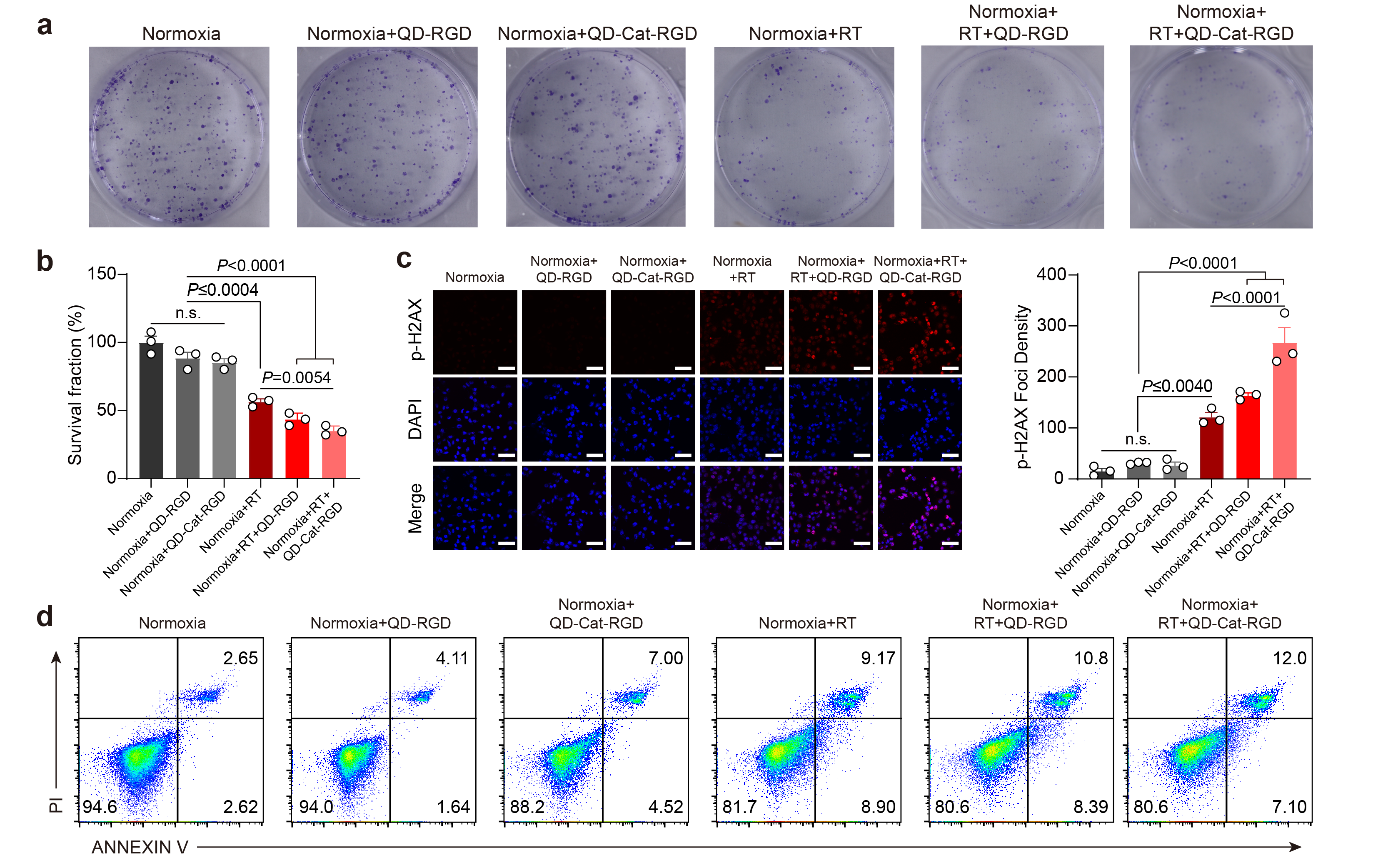


**Supplementary Fig. 5** QD-Cat-RGD as a radiosensitizer under normoxic conditions. 4T1 cells were incubated under normoxic conditions (37 °C, 20% O_2_, 5% CO_2_) and irradiated with X-ray doses of 6 Gy in vitro. **a** Representative photographs of stained colonies of 4T1 cells treated with normoxic, normoxic + QD-RGD, normoxic + QD-Cat-RGD, normoxic + RT (6 Gy), normoxic + RT + QD-RGD (6 Gy) or normoxic + RT + QD-Cat-RGD (6 Gy) after 7 days. **b** Histogram plot of the survival fraction of 4T1 cells with different treatments. **c** Immunofluorescence staining of p-H2AX (Ser139) and quantitative analysis of foci density of foci per cell at 4 h after different treatments. Scale bars = 25 μm. The images of immunofluorescence staining (**c**) were representative of those generated from three independent samples each group. **d** Apoptosis analysis measured by flow cytometry of 4T1 cells at 24 h after different treatments. All data are shown as the mean ± s.e.m. (n = 3) and n represents the number of independent samples. Statistical significance was calculated via one-way ANOVA with Tukey’s multiple comparisons test (**b, c**). n.s., not significant. Source data are provided as a Source Data file.


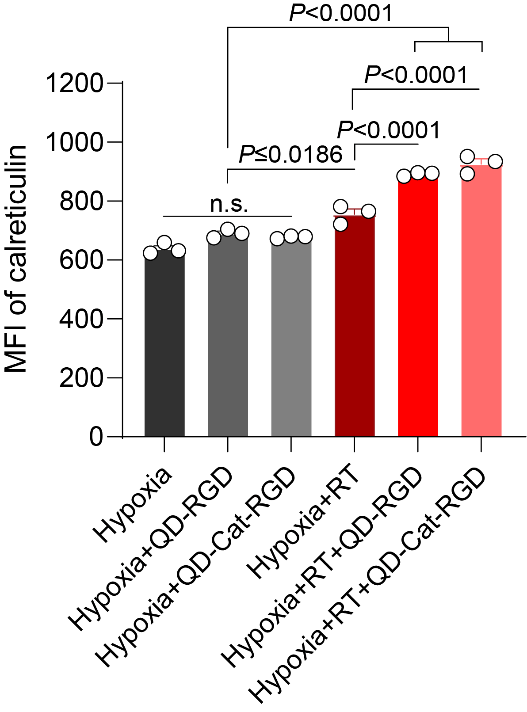


**Supplementary Fig. 6** Flow cytometry analysis of surface calreticulin expression in 4T1 cells with different treatments after 24 h. All data are shown as the mean ± s.e.m. of three replicates. Statistical significance was calculated via one-way ANOVA with Tukey’s multiple comparisons test. n.s., not significant. MFI, median fluorescence intensity. Source data are provided as a Source Data file.


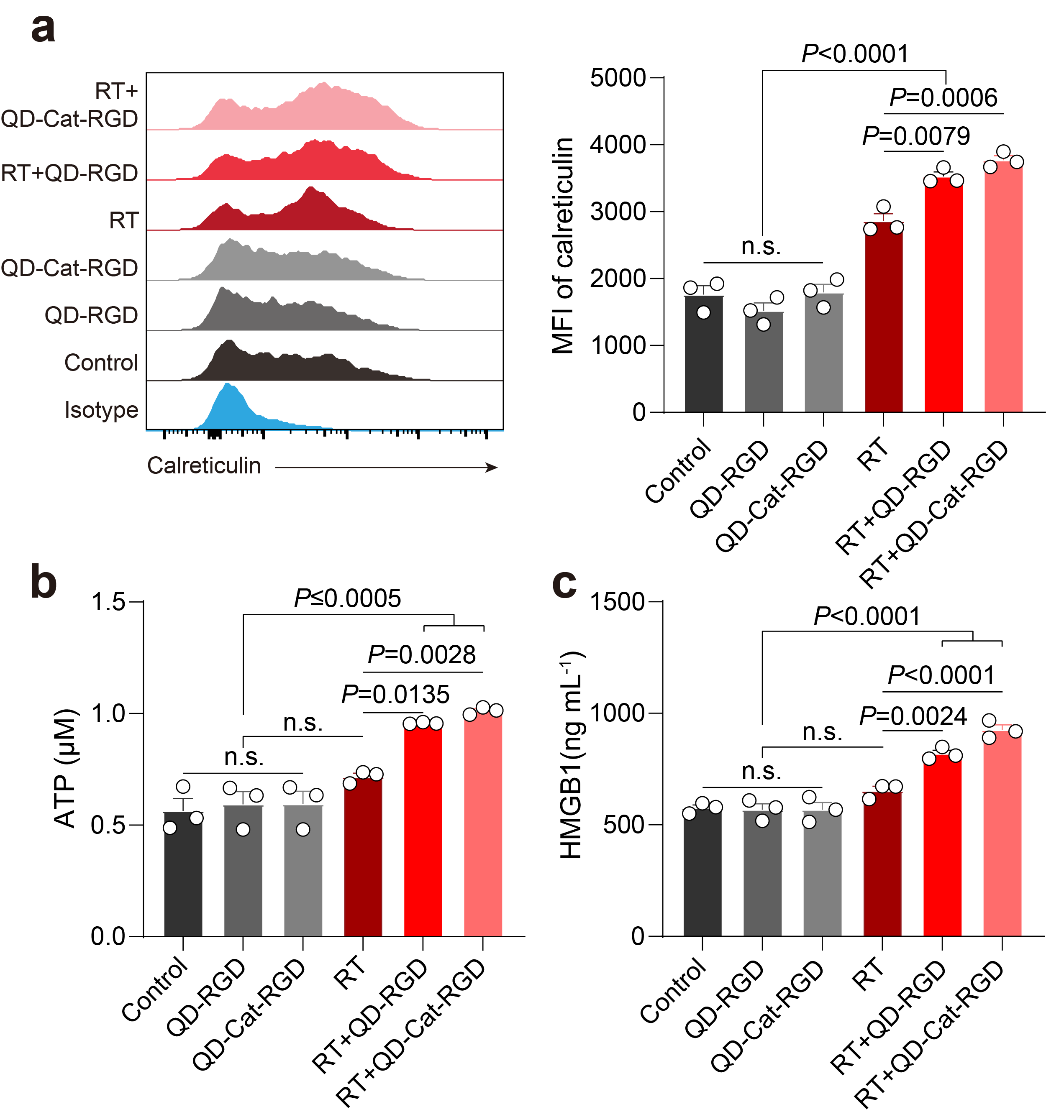


**Supplementary Fig. 7 a** Flow cytometry analysis of surface calreticulin expression in tumor tissue cells with different treatments after 24 h. **b** Histogram plot of ATP concentration in tumor tissue with different treatments after 24 h. **c** Histogram plot chart of HMGB1 concentration in tumor tissue with different treatments after 24 h. All data are shown as the mean ± s.e.m. (n = 3) and n represents the number of independent samples. Statistical significance was calculated via one-way ANOVA with Tukey’s multiple comparisons test. n.s., not significant. MFI, median fluorescence intensity. Source data are provided as a Source Data file.


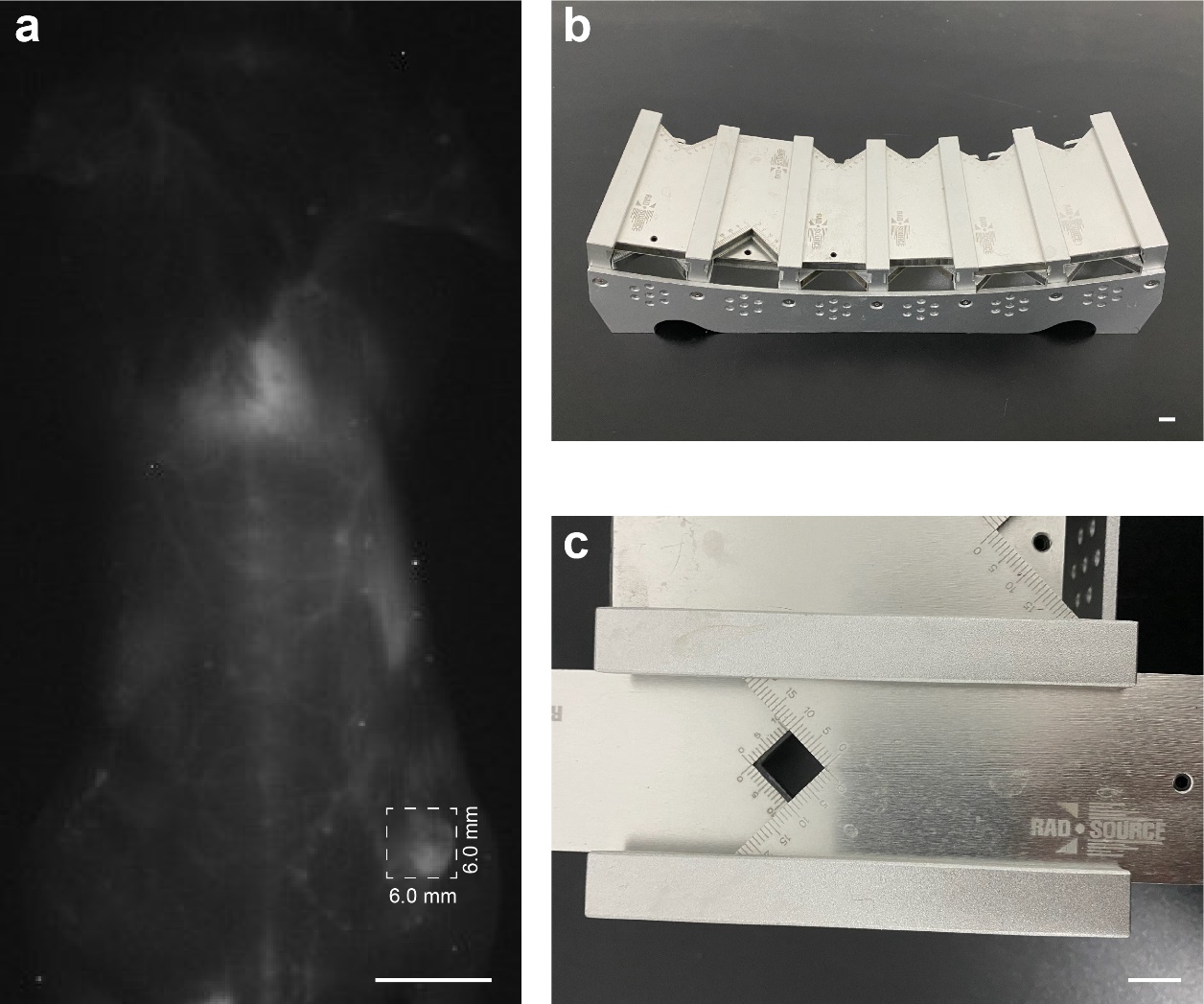


**Supplementary Fig. 8 a** The location and range of tumor were clearly demonstrated in the NIR-IIb fluorescence imaging after QD-Cat-RGD injection. Scale bars = 1 cm. **b, c** The special lead shield for tumor-bearing mouse to localize the range of RT determined by the NIR-IIb imaging. Scale bars = 1 cm. The dosimetry validation of RT was made by the Accu-Dose+ stream-line diagnostic solution (Radcal). The fluorescent images in the NIR-IIb window (**a**) were representative of those generated from five mice treated with RT+QD-Cat-RGD. Source data are provided as a Source Data file.


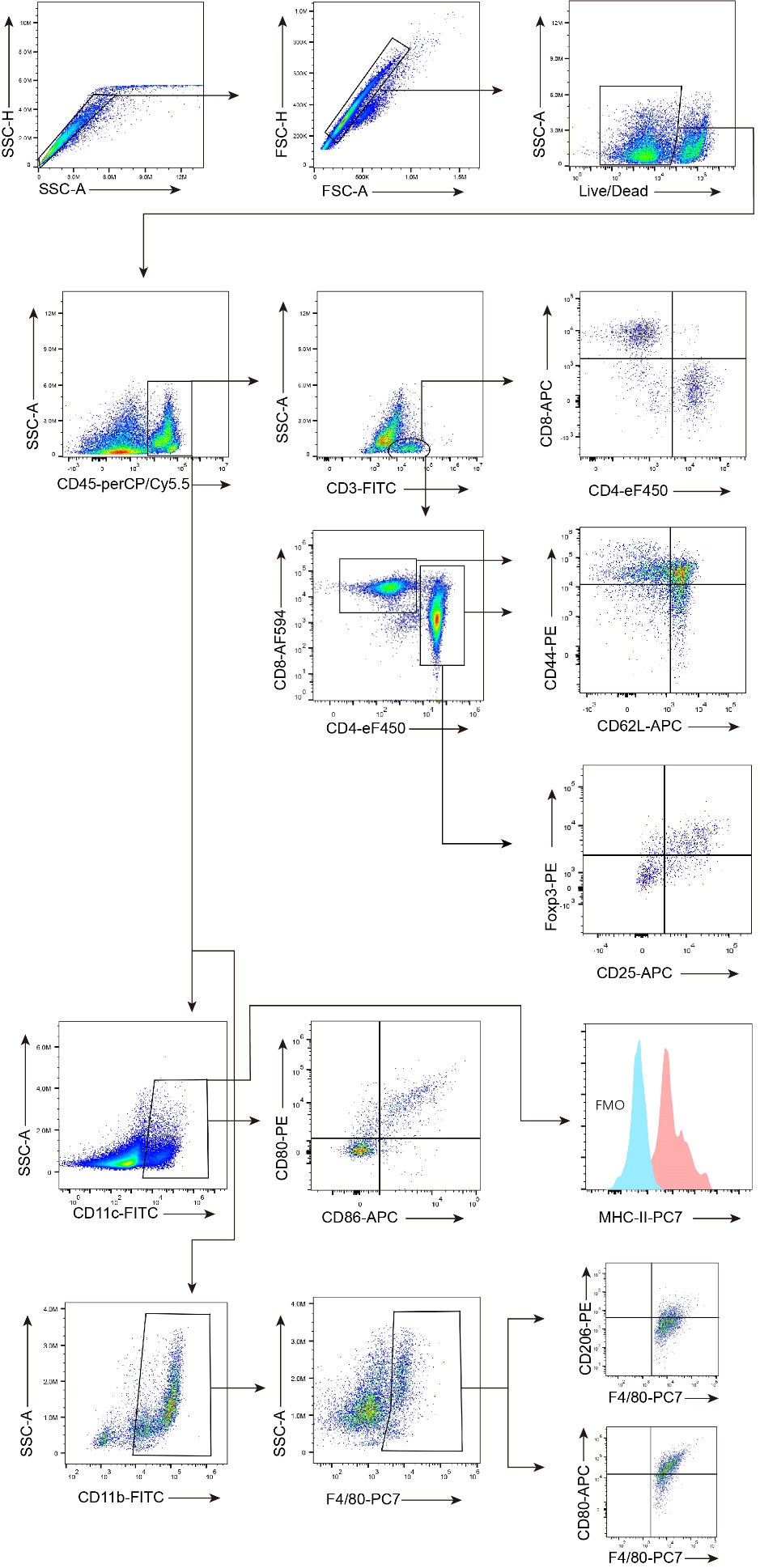


**Supplementary Fig. 9** Gating strategy used to identify the CD45^+^ cells from tumor tissue and tumor draining lymph node (TDLN).


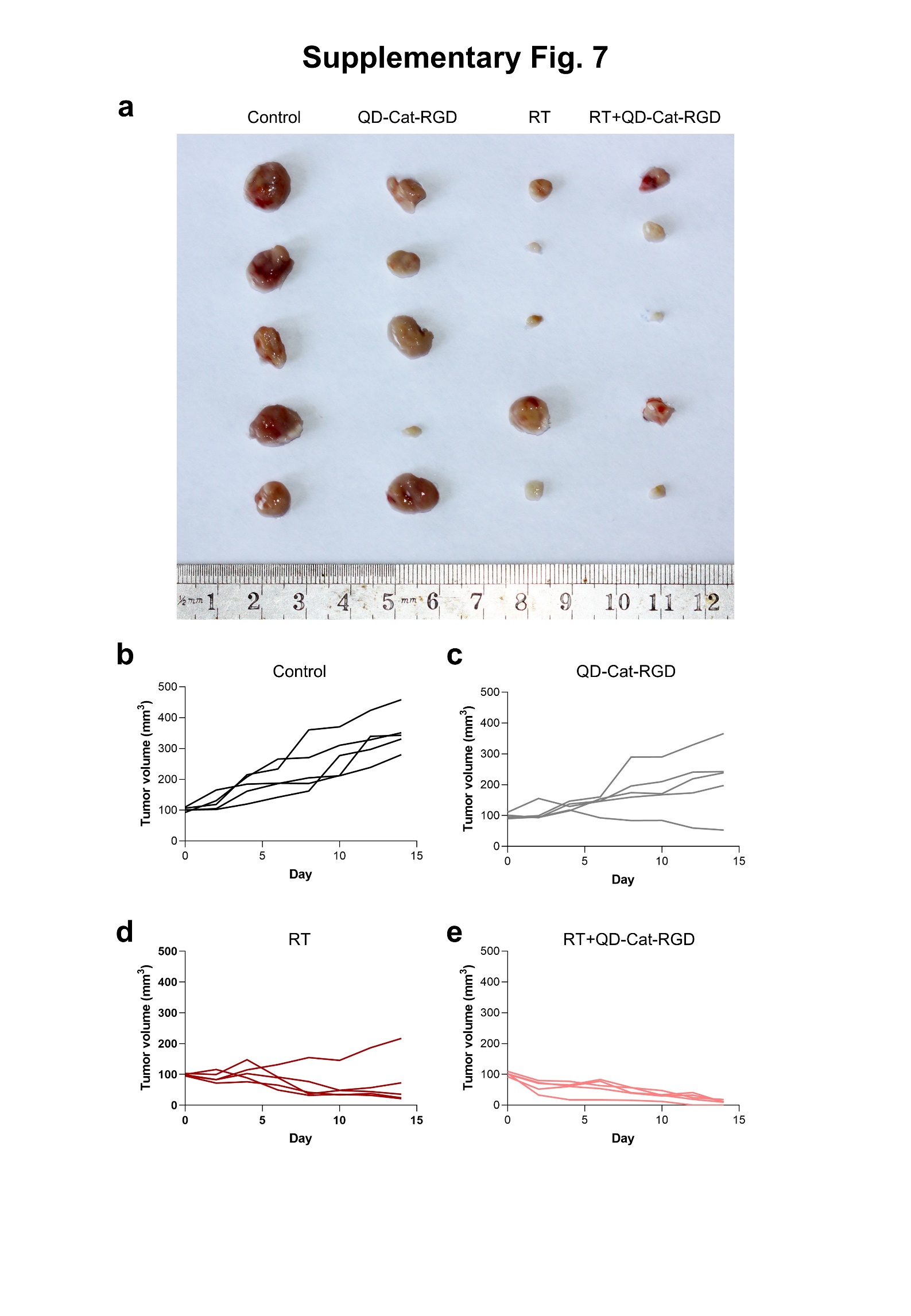


**Supplementary Fig. 10 a** Photograph of tumor tissue harvested from treated 4T1 tumor-bearing mice. **b-e** Tumor growth curve of 4T1 tumor-bearing mice in control and treated groups (n = 5 per group) and n represents the number of independent animals. Source data are provided as a Source Data file. Supplementary Fig. 10, related to Figure 4b.


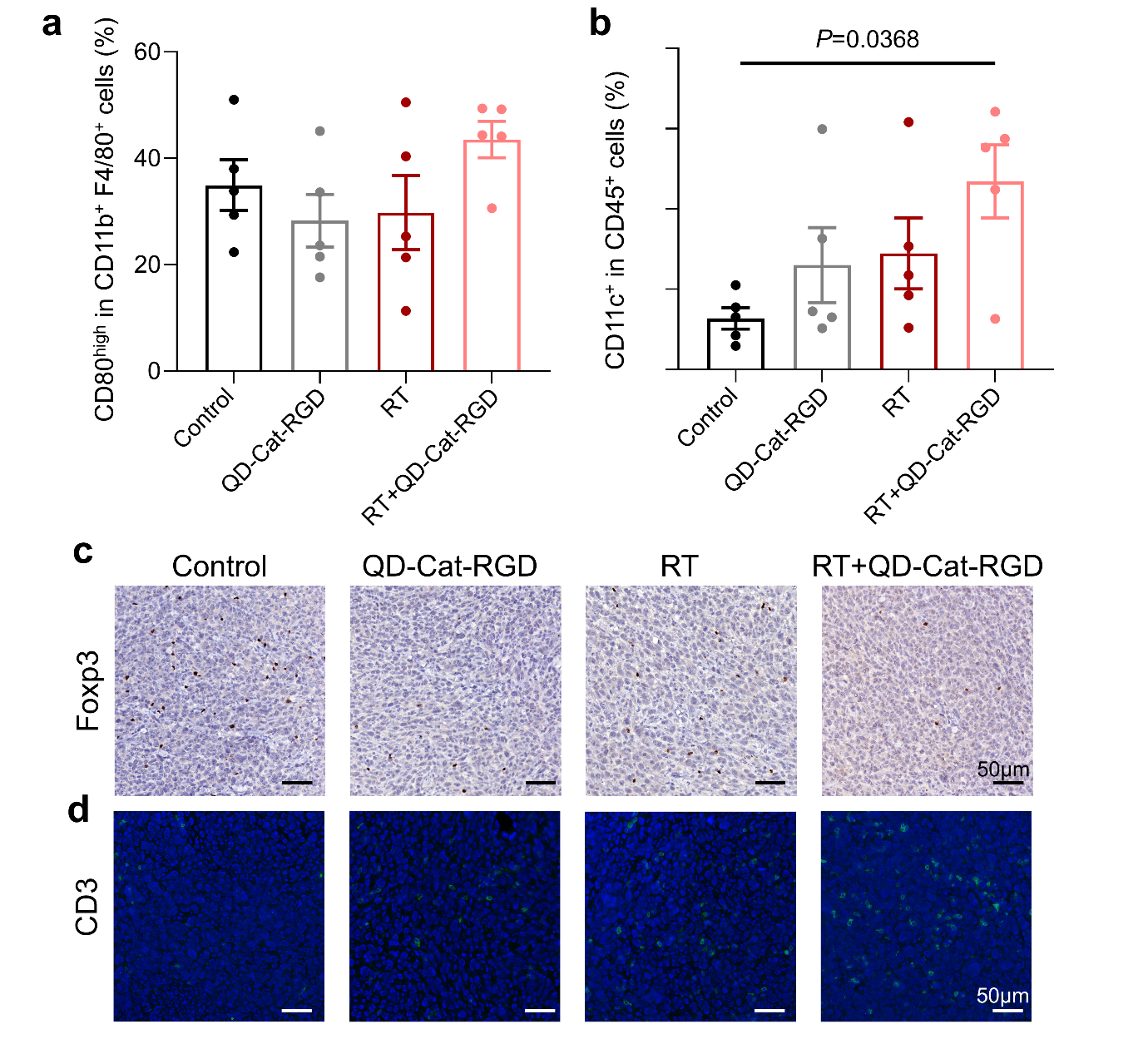


**Supplementary Fig. 11** **a** Relative quantification of M1-like (CD80^high^) macrophages gating on F4/80^+^CD11b^+^CD45^+^ cells in tumors. **b** Relative quantification of CD11c^+^ DC cells gating on CD45^+^ cells in tumors. The data (**a**, **b**) are presented as the mean ± s.e.m. (n = 5 per group) and n represents the number of independent animals. Statistical significance was calculated via one-way ANOVA with Tukey’s multiple comparisons test. **c** Representative immunohistochemistry (IHC) images of tumors showing Foxp3^+^ Treg cells. Scale bars = 50 μm. **d** Representative immunofluorescence images of tumors showing CD3^+^ T cells. Scale bars = 50 μm. The images of immunostaining (**c**, **d**) were representative of those generated from three mice each group. Source data are provided as a Source Data file.


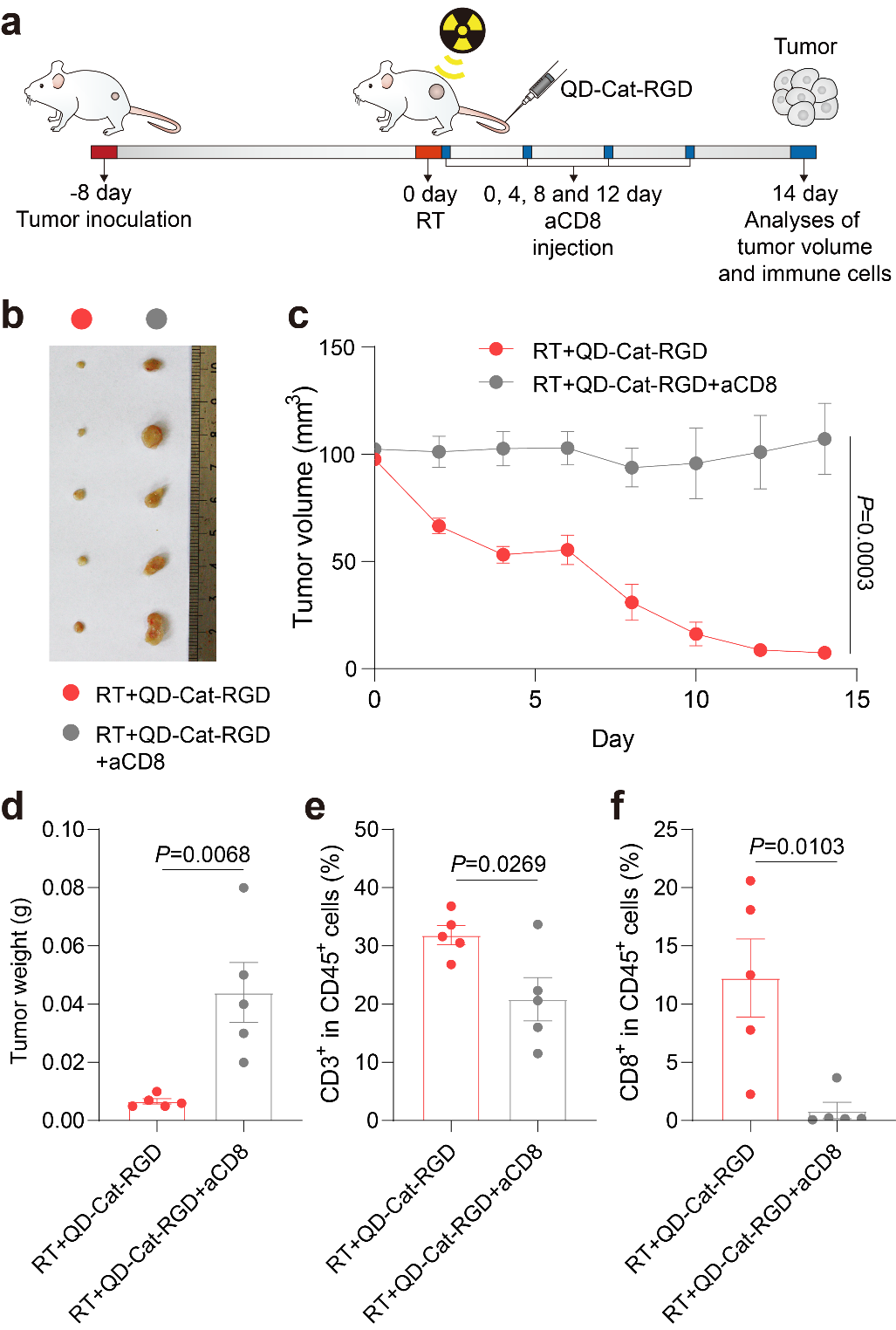


**Supplementary Fig. 12 QD-Cat-RGD-based RT reducing the effect inhibiting tumor growth when depleting the CD8^+^ cytotoxic T cells. a** Schematic showing the experiment using QD-Cat-RGD-based RT combined with antibodies against CD8 (aCD8) to treat mice bearing 4T1 tumors. BALB/c mice (n = 5 per group) were implanted subcutaneously with 7× 10^5^ 4T1 mammary carcinoma cells in the right hind flanks. When the tumor volumes were 100 mm^3^, mice received different treatments. 4T1 tumors were harvested on day 14. **b** Photographs of all tumors from individual mice. **c** Tumor growth curves in different groups. **d** Histogram plot of tumor weight in different groups. **e** Quantification of flow cytometric analysis of CD3^+^ T cells gating on CD45^+^ in the tumors of the different groups. **f** Quantification of flow cytometric analysis of CD8^+^CD3^+^ T cells gating on CD45^+^ in tumors of the different groups. All data are presented as the mean ± s.e.m. (n = 5 per group) and n represents the number of independent animals. Statistical significance was calculated via two-tailed Student’s *t* test. Source data are provided as a Source Data file.


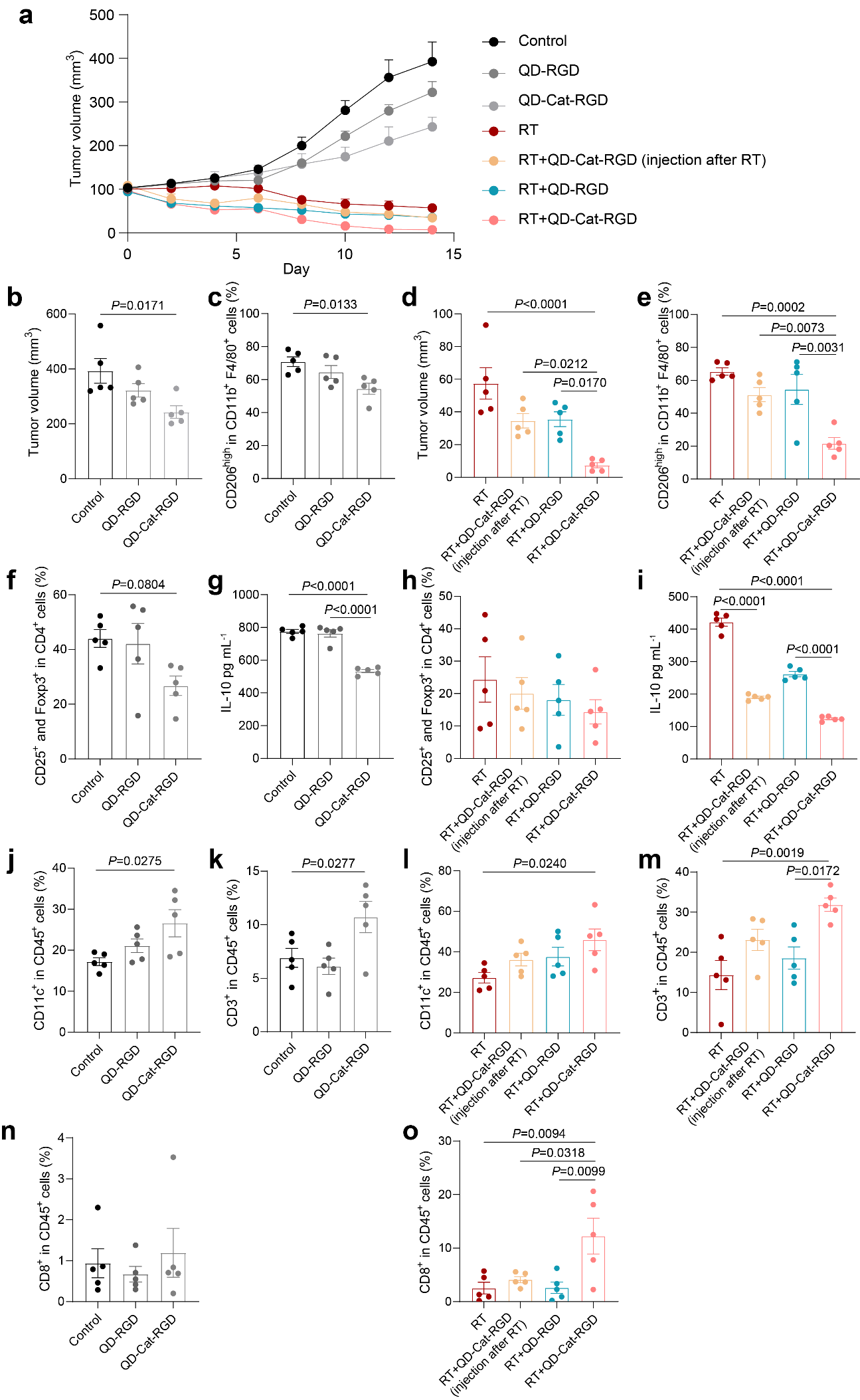


**Supplementary Fig. 13** BALB/c mice (n = 5 per group) were implanted subcutaneously with 7× 10^5^ 4T1 mammary carcinoma cells in the right hind flanks. When the tumor volumes reached approximately 100 mm^3^, mice were intravenously injected with a single dose of QD-Cat-RGD (150μL, 2 mg mL^-1^), QD-RGD (150μL, 2 mg mL^-1^) or phosphate-buffered saline (PBS,150 μL). Tumors were harvested on day 14. **a** Tumor growth curves in different groups. **b, d** Histogram plot of tumor volume in non-RT groups (**b**) and RT groups (**d**). **c, e** The quantification of M2-like macrophages (CD206^high^) gating on F4/80^+^CD11b^+^CD45^+^ cells in non-RT groups (**c**) and RT groups (**e**). **f, h** The quantification of Tregs (CD25^+^ and Foxp3^+^) gating on CD4^+^CD3^+^CD45^+^ cells in non-RT groups (**f**) and RT groups (**h**). **g, i** Cytokine level of IL-10 measured by ELISA in non-RT groups (**g**) and RT groups (**i**). **j, l** The quantification of DC (CD11c^+^) gating on CD45^+^ cells in non-RT groups (**j**) and RT groups (**l**). **k, m** The quantification of T cells (CD3^+^) gating on CD45^+^ cells in non-RT groups (**k**) and RT groups (**m**). **n, o** The quantification of cytotoxic T cells (CD8^+^CD3^+^) gating on CD45^+^ cells in non-RT groups (**n**) and RT groups (**o**). All data are presented as the mean ± s.e.m. (n = 5 per group) and n represents the number of independent animals. Statistical significance was calculated via one-way ANOVA with Tukey’s multiple comparisons test. Source data are provided as a Source Data file.


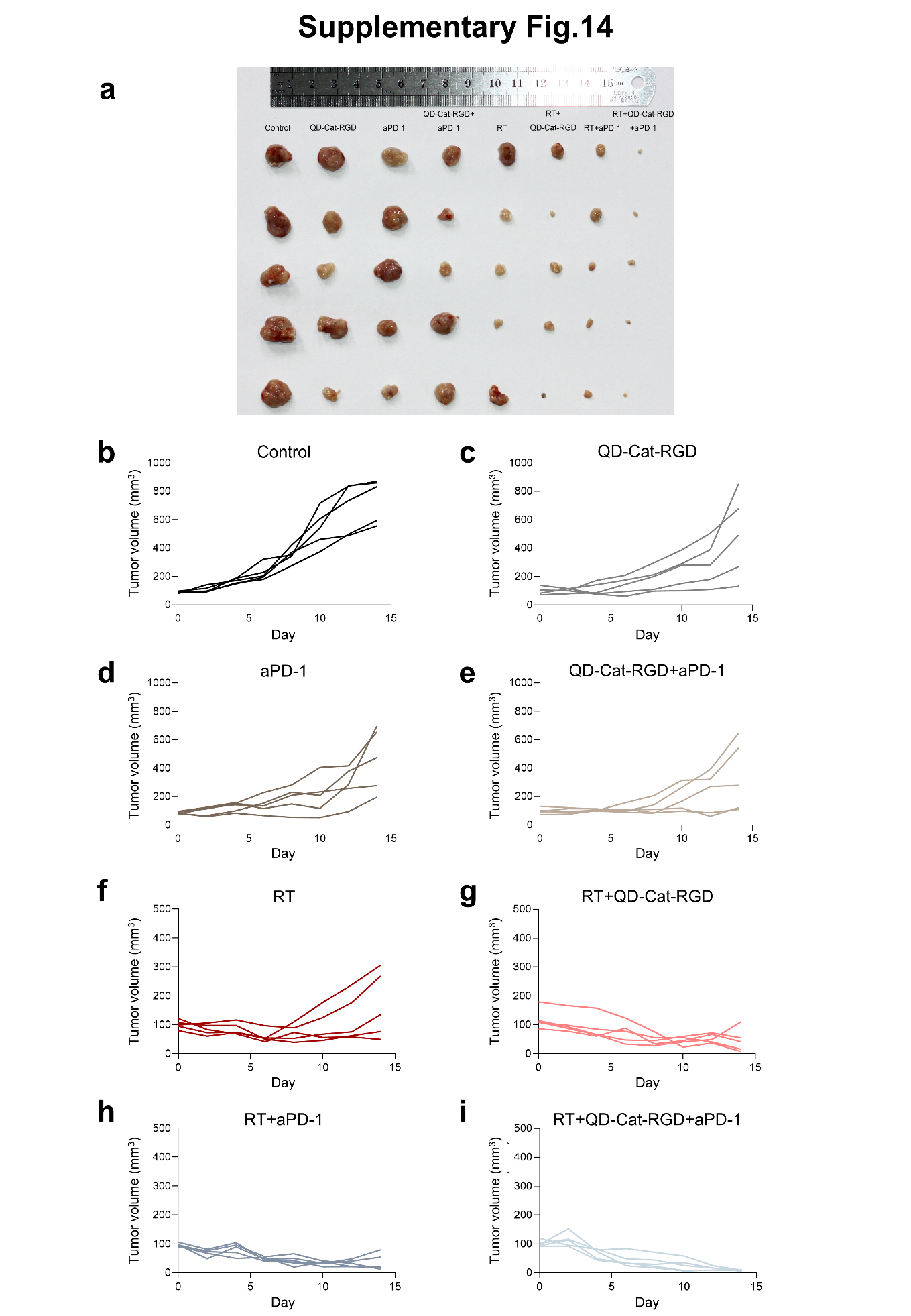


**Supplementary Fig. 14** **a** Photograph of primary tumor tissue harvested from treated 4T1 tumor-bearing mice. **b-i** Primary tumor growth curve of 4T1 tumor-bearing mice in control and treated groups (n = 5 per group) and n represents the number of independent animals. Supplementary Fig. 14, related to Figure 6b. Source data are provided as a Source Data file.


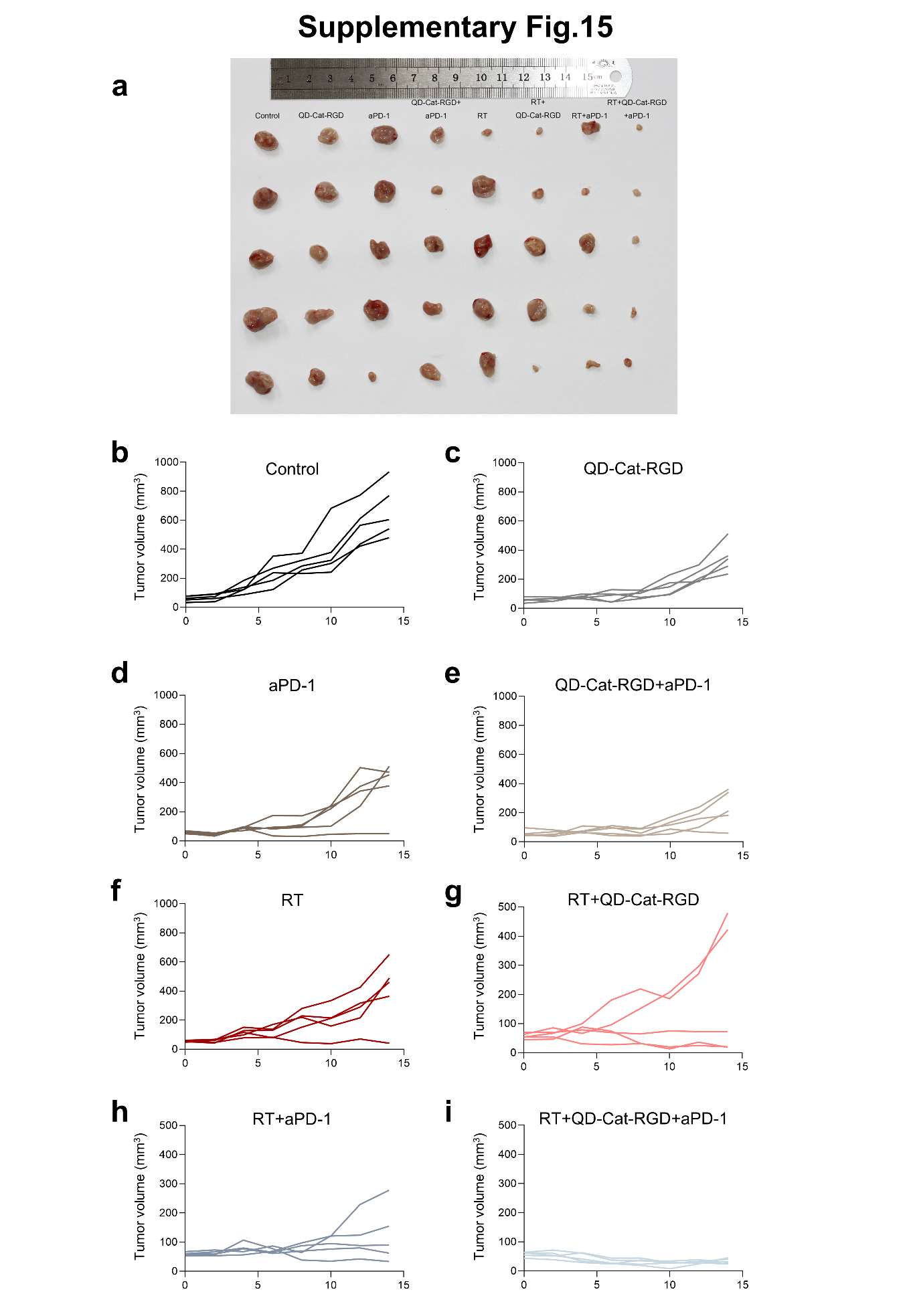


**Supplementary Fig. 15 a** Photograph of distant tumor tissue harvested from treated 4T1 tumor-bearing mice. **b-i** Distant tumor growth curve of 4T1 tumor-bearing mice in control and treated groups (n = 5 per group) and n represents the number of independent animals. Supplementary Fig. 15, related to Figure 6c. Source data are provided as a Source Data file.


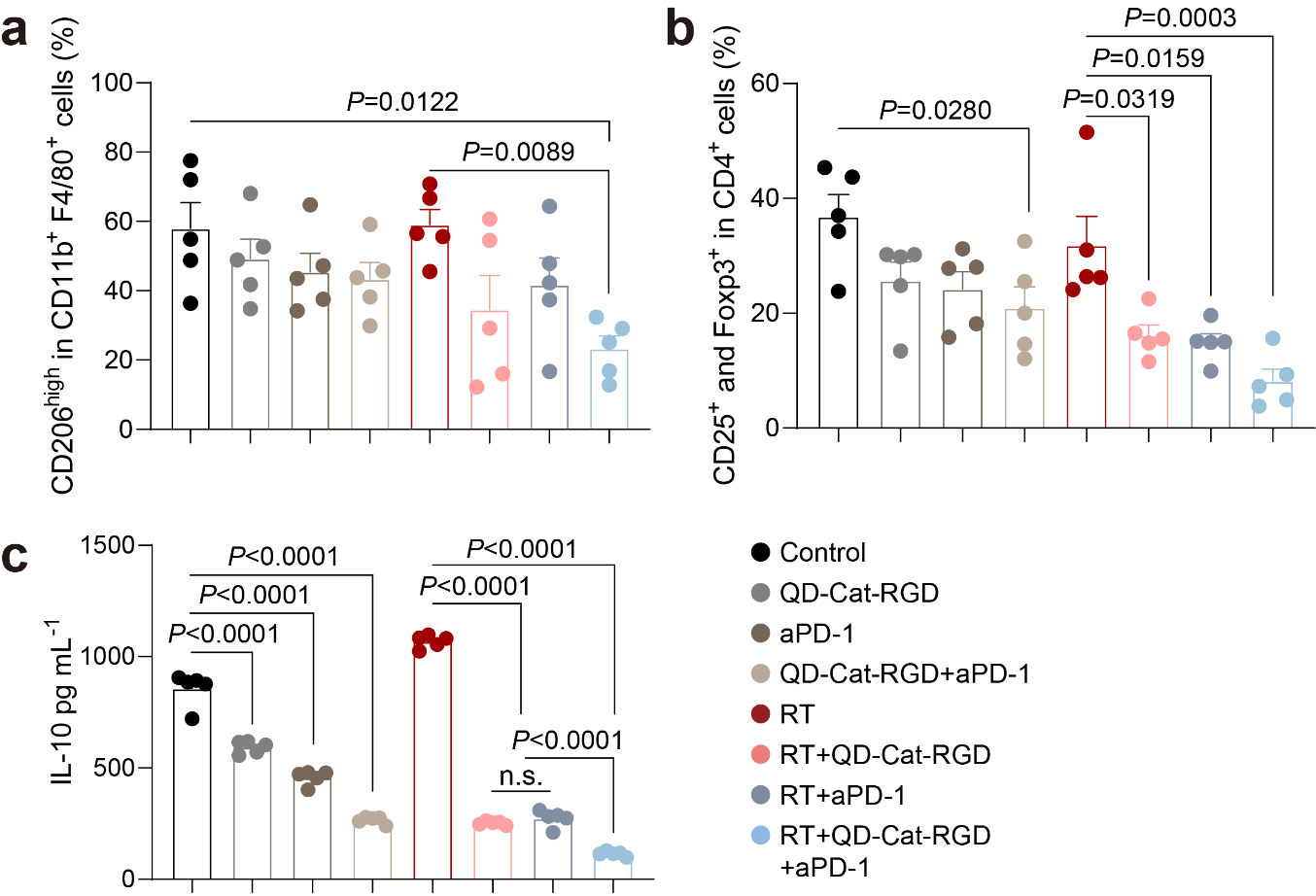


**Supplementary Fig. 16** **a** The quantification of M2-like macrophages (CD206^high^) gating on F4/80^+^CD11b^+^CD45^+^ cells in distant tumors after different treatment. **b** The quantification of Tregs (CD25^+^ and Foxp3^+^) gating on CD4^+^CD3^+^CD45^+^ cells in distant tumors after different treatment. **c** Cytokine level of IL-10 in distant tumors after different tumors measured by ELISA. All data are presented as the mean ± s.e.m. (n = 5) and n represents the number of independent animals. Statistical significance was calculated via one-way ANOVA with Tukey’s multiple comparisons test. Source data are provided as a Source Data file.


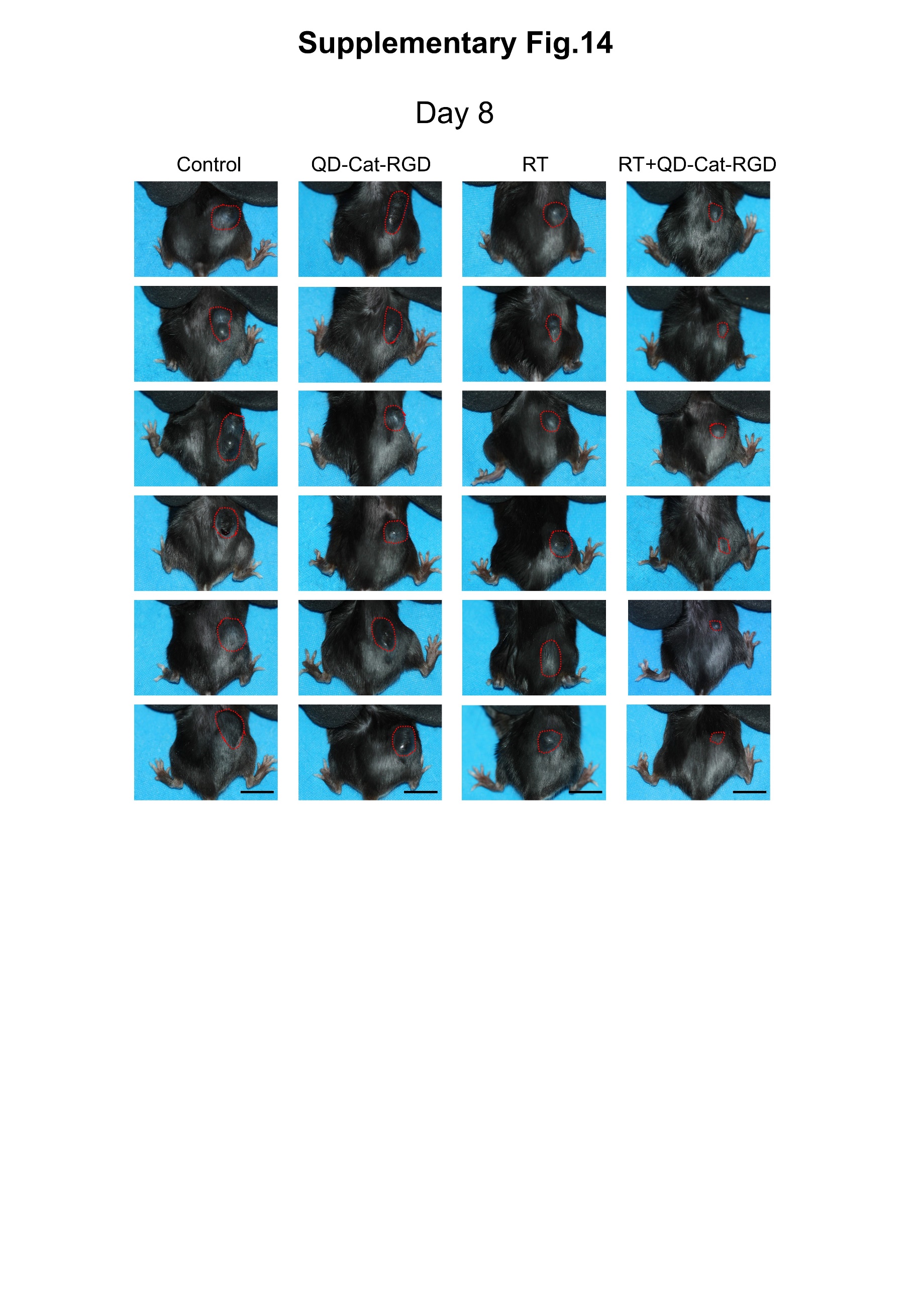


**Supplementary Fig. 17** Photographs showing the tumor volume of B16F10 tumor-bearing C57BL/6 mice after different treatments on day 8 (n = 6 per group) and n represents the number of independent animals. Scale bar = 1cm.


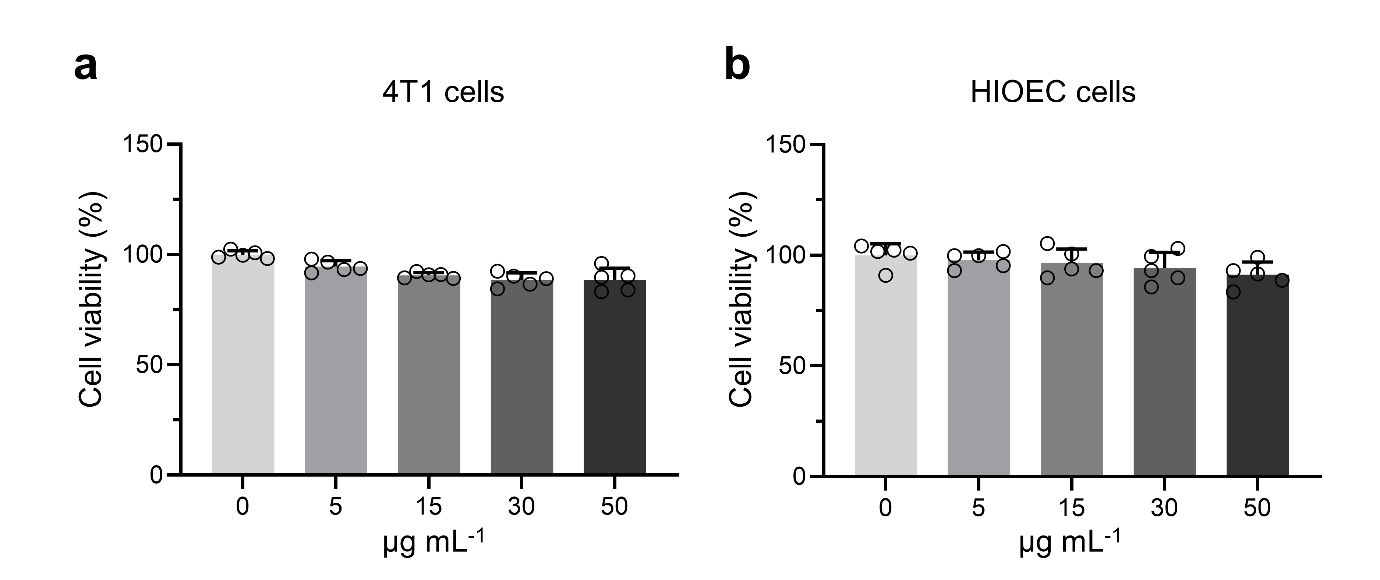


**Supplementary Fig. 18** The cytotoxicity of QD-Cat-RGD measured by Cell Counting Kit-8 (CCK8) revealed that the cell viability of 4T1 (**a**) and HIOEC (**b**) cells. All data are shown as the mean ± s.d. (n = 5) and n represents the number of independent samples. Source data are provided as a Source Data file.


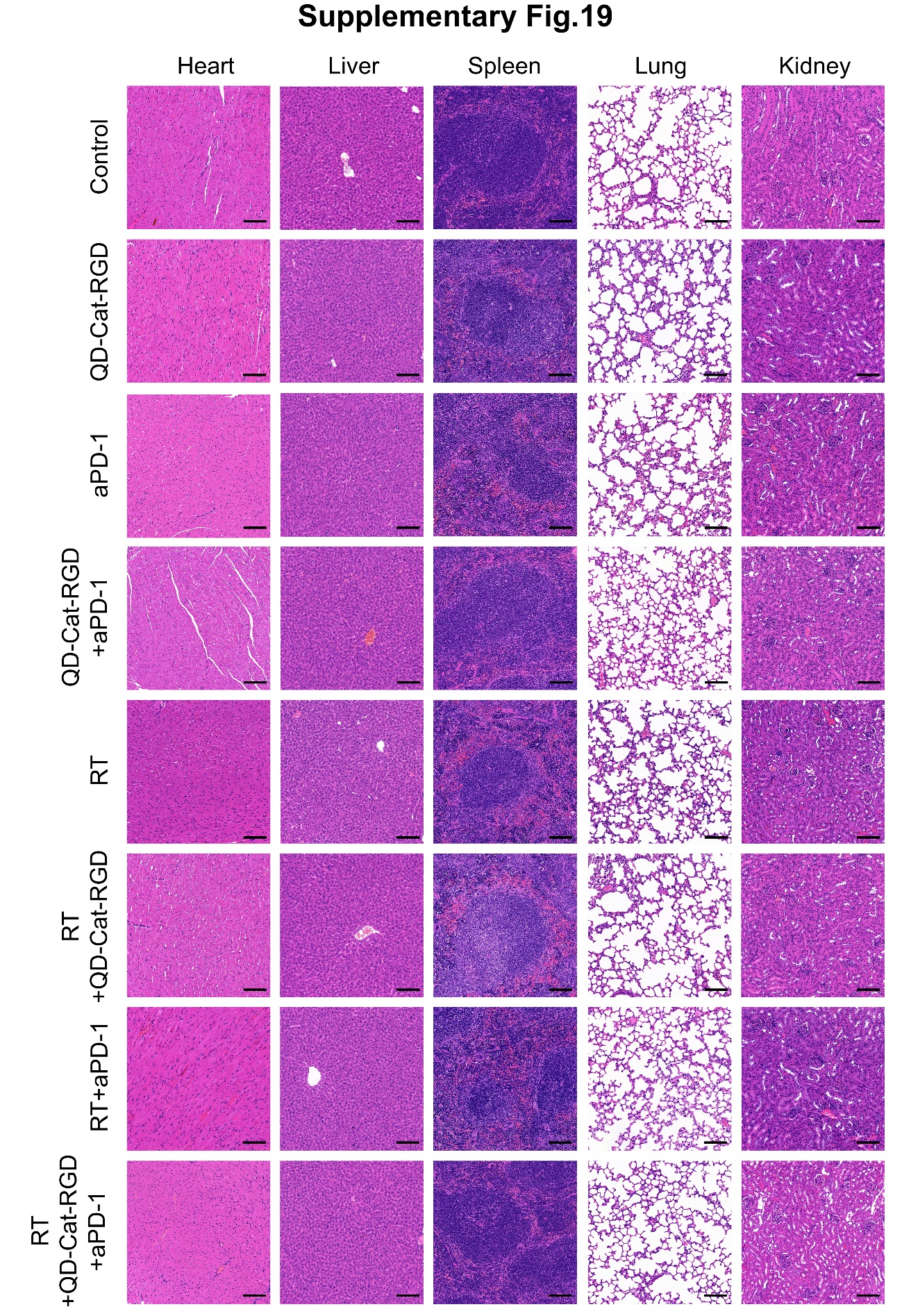


**Supplementary Fig. 19** Hematoxylin and eosin staining (H&E staining) results of heart, liver, spleen, lungs, and kidneys in treated mice showed no abnormality in cellar morphology compared with control mice. Scale bars = 100 μm. The images of H&E staining were representative of those generated from three mice each group.

| Parameter | Unit | Control (Mean ± s.d.) | QD-Cat-RGD (Mean ± s.d.) |
| --- | --- | --- | --- |
| WBC | 10^9^ L^-1^ | 4.033 ± 4.933 | 2.433 ± 0.680 |
| RBC | 10^12^ L^-1^ | 11.907 ± 0.659 | 9.567 ± 1.124 |
| MCV | fL | 48.433 ± 0.386 | 46.967 ± 0.680 |
| MCH | pg | 15.667 ± 0.236 | 14.9 ± 0.294 |
| MCHC | g L^-1^ | 324.667 ± 7.318 | 317.667 ± 2.055 |
| RDW | CV% | 14.867 ± 0.544 | 17.367 ± 1.569 |
| PLT | 10^9^ L^-1^ | 543.333 ± 89.235 | 496.333 ± 214.025 |
| MPV | fL | 6.000 ± 0.082 | 6.200 ± 0.163 |
| PCT | % | 0.326 ± 0.057 | 0.304 ± 0.128 |
| ALT | U L^-1^ | 56.590 ± 3.310 | 65.956 ± 9.299 |
| AST | U L^-1^ | 189.674 ± 60.536 | 214.651 ± 45.896 |
| UREA | mmol L^-1^ | 21.282 ± 1.148 | 23.523 ± 1.165 |
| CREA | μmol L^-1^ | 16.234 ± 0.614 | 16.929 ± 4.326 |

**Supplementary Table 1** Routine blood test and blood biochemical test of mice in the control and QD-Cat-RGD injection groups. WBC: white blood cell; RBC: red blood cell; MCV: mean corpuscular volume, MCH: mean corpuscular hemoglobin; MCHC: mean corpuscular hemoglobin concentration; RDW: red cell distribution width; PLT: platelets; MPV: mean platelet volume; PCT: Platelet cubic measure distributing width; ALT: Alanine aminotransferase; AST: Aspartate aminotransferase; UREA: carbamide; CREA: Creatinine. Standard deviations (s.d.) were calculated based on data from three mice in each group. Source data are provided as a Source Data file.
