# Supplementary material for: Theranostic near-infrared-IIb emitting nanoprobes for promoting immunogenic radiotherapy and abscopal effects against cancer metastasis
Source: Nat Commun. 2021 Dec 9;12:7149. doi: 10.1038/s41467-021-27485-0 (PMC8660774; doi:10.1038/s41467-021-27485-0)
Supplement: Supplementary file 2 — Reporting Summary [file 41467_2021_27485_MOESM2_ESM.pdf]

nature research

Corresponding author(s): Ran Cui, Mingxi Zhang, Zhi-Jun Sun

Last updated by author(s): Nov 19, 2021

# Reporting Summary

Nature Research wishes to improve the reproducibility of the work that we publish. This form provides structure for consistency and transparency in reporting. For further information on Nature Research policies, see our Editorial Policies and the Editorial Policy Checklist.

## Statistics

For all statistical analyses, confirm that the following items are present in the figure legend, table legend, main text, or Methods section.

| n/a | Confirmed | |
|---|---|---|
| ☐ | ☒ | The exact sample size ($n$) for each experimental group/condition, given as a discrete number and unit of measurement |
| ☐ | ☒ | A statement on whether measurements were taken from distinct samples or whether the same sample was measured repeatedly |
| ☐ | ☒ | The statistical test(s) used AND whether they are one- or two-sided<br>*Only common tests should be described solely by name; describe more complex techniques in the Methods section.* |
| ☐ | ☒ | A description of all covariates tested |
| ☐ | ☒ | A description of any assumptions or corrections, such as tests of normality and adjustment for multiple comparisons |
| ☐ | ☒ | A full description of the statistical parameters including central tendency (e.g. means) or other basic estimates (e.g. regression coefficient) AND variation (e.g. standard deviation) or associated estimates of uncertainty (e.g. confidence intervals) |
| ☐ | ☒ | For null hypothesis testing, the test statistic (e.g. $F$, $t$, $r$) with confidence intervals, effect sizes, degrees of freedom and $P$ value noted<br>*Give P values as exact values whenever suitable.* |
| ☒ | ☐ | For Bayesian analysis, information on the choice of priors and Markov chain Monte Carlo settings |
| ☒ | ☐ | For hierarchical and complex designs, identification of the appropriate level for tests and full reporting of outcomes |
| ☒ | ☐ | Estimates of effect sizes (e.g. Cohen's $d$, Pearson's $r$), indicating how they were calculated |

*Our web collection on statistics for biologists contains articles on many of the points above.*

## Software and code

Policy information about availability of computer code

| | |
|---|---|
| Data collection | CytExpert software v. 2.3(Beckman), Image Studio software v. 5.2(Li-Cor Biosciences), ImageScope software v. 12.3.2 (Leica), FV10-ASW v. 4.0 software (Olympus), Living Image software (PerkinElmer), LightField software v. 6.4 (Princeton Instruments). |
| Data analysis | Softwares used for analysis include GraphPad Prism v. 8.0 (GraphPad Software), FlowJo v. 10 (TreeStar), Living image software (PerkinElmer), Aperio ImageScope v. 12.3.2 (Leica), Image-Pro Plus v. 6.0 (Media Cybernetics), LightField software v. 6.4 (Princeton Instruments), Excel 2016 (Microsoft), Image Studio software v. 5.2 (Li-Cor Biosciences), CaseViewer v. 2.4 (3DHISTECH), PKSolver software v. 2.0. |

For manuscripts utilizing custom algorithms or software that are central to the research but not yet described in published literature, software must be made available to editors and reviewers. We strongly encourage code deposition in a community repository (e.g. GitHub). See the Nature Research guidelines for submitting code & software for further information.

## Data

Policy information about availability of data

All manuscripts must include a data availability statement. This statement should provide the following information, where applicable:

- Accession codes, unique identifiers, or web links for publicly available datasets
- A list of figures that have associated raw data
- A description of any restrictions on data availability

The data generated in this study within the Article and Supplementary Information are available and provided in the Source Data file. Source data are provided with this paper.

# Field-specific reporting

Please select the one below that is the best fit for your research. If you are not sure, read the appropriate sections before making your selection.

- [x] Life sciences
- [ ] Behavioural & social sciences
- [ ] Ecological, evolutionary & environmental sciences

For a reference copy of the document with all sections, see nature.com/documents/nr-reporting-summary-flat.pdf

# Life sciences study design

All studies must disclose on these points even when the disclosure is negative.

| | |
|---|---|
| Sample size | Although no sample size calculation was performed, sample sizes for the in vivo experiments are similar to those generally employed and accepted in the field (Nat Nanotechnol 14(1):89-97 (2019); Nat Biotechnol 37(11):1322-1331 (2019) and were sufficient to support our conclusions with statistical significance. Sample sizes for the in vitro experiments are also based on previous work (Nat Commun 4;12(1):6371 (2021)). |
| Data exclusions | No data were excluded. |
| Replication | All results obtained in this study were successfully replicated. Except animal experiments, we have performed the experiments shown in our manuscript more than twice. Exact numbers of biologically independent repetitions are stated in the manuscript. |
| Randomization | Mice were allocated randomly to each experimental group. |
| Blinding | In vivo experiments were performed unblinded due to the requirements and regulations of the SPF Animal Laboratory of School and Hospital of Stomatology of Wuhan University. Bioluminescence imaging were conducted by an independent operator, who was unaware of the treatment conditions. |

# Reporting for specific materials, systems and methods

We require information from authors about some types of materials, experimental systems and methods used in many studies. Here, indicate whether each material, system or method listed is relevant to your study. If you are not sure if a list item applies to your research, read the appropriate section before selecting a response.

## Materials & experimental systems

| n/a | Involved in the study | |
|---|---|---|
| [ ] | [x] | Antibodies |
| [ ] | [x] | Eukaryotic cell lines |
| [x] | [ ] | Palaeontology and archaeology |
| [ ] | [x] | Animals and other organisms |
| [x] | [ ] | Human research participants |
| [x] | [ ] | Clinical data |
| [x] | [ ] | Dual use research of concern |

## Methods

| n/a | Involved in the study | |
|---|---|---|
| [x] | [ ] | ChIP-seq |
| [ ] | [x] | Flow cytometry |
| [x] | [ ] | MRI-based neuroimaging |

## Antibodies

Antibodies used

The following primary antibodies were used for blocking. They are listed as antigen first, followed by supplier and clone/catalog number as applicable.
1) anti-mouse PD-1 (aPD-1), 200 μg per mouse, Bioxcell, Clone RMP1-14, Cat BE0146, ;
2) anti-mouse CD8 (aCD8), 200 μg per mouse, Bioxcell, Clone 53-6.7, Cat BE0001-1.

The following primary antibodies were used for western blotting. They are listed as antigen first, followed by supplier, catalog number as applicable.
1) anti-GAPDH, 1:5000, Proteintech, Cat HRP-60004;
2) anti-HIF-1α, 1:500, Novus Biologicals, Cat NB-100-105.

The following primary antibodies were used for immunohistochemistry (IHC) and immunofluorescence. They are listed as antigen first, followed by supplier, catalog number as applicable.
1) anti-CD3ε, 1:200, Cell Signaling Technology, Cat 99940;
2) anti-CD8α, 1:400, Cell Signaling Technology, Cat 98941;
3) anti-Foxp3, 1:400, Cell Signaling Technology, Cat 12653;
4) anti-granzyme B, 1:200, Cell Signaling Technology, Cat 44153;
5) anti-HIF-1α, 1:150, Novus Biologicals, Cat NB-100-105;

6) anti-phospho-histone H2AX (Ser139/Tyr142), 1:100, Cell Signaling Technology, Cat 5438;
7) anti-calreticulin, 1:400, Cell Signaling Technology, Cat 12238;
8) anti-HMGB1, 1:400, Abcam, Cat ab18256;
9) HRP-conjugated secondary antibody (anti-mouse), undiluted, Cell Signaling Technology, Cat 8125;
10) HRP-conjugated secondary antibody (anti-rabbit), undiluted, Cell Signaling Technology, Cat 8114;
11) Fluorescence conjugated secondary antibody (anti-rabbit, DyLight 488), 1:500, Abbkine, Cat A23220;
12) Fluorescence conjugated secondary antibody (anti-rabbit, DyLight 594), 1:500, Abbkine, Cat A23430.

The following primary antibodies were used for flow cytometry. They are listed as antigen first, followed by supplier and clone/ catalog number as applicable.
1) anti-mouse CD45, 1:500, eBioscience, perCP-Cyanine5.5, Clone 30-F11, Cat 45-0451-82;
2) anti-mouse CD3e, 1:500, eBioscience, FITC, Clone 145-2C11, Cat 11-0031-82;
3) anti-mouse CD4, 1:500, eBioscience, eFluor 450, Clone RM4-5, Cat 48-0042-82;
4) anti-mouse CD8a, 1:500, Biolegend, APC, Clone 53-6.7, Cat 100712;
5) anti-mouse CD8a, 1:500, Biolegend, Alexa Fluor 594, Clone 53-6.7, Cat 100758;
6) anti-mouse CD11b, 1:500, Biolegend, FITC, Clone M1/70, Cat 101206;
7) anti-mouse F4/80, 1:500, eBioscience, PE-Cyanine7, Clone BM8, Cat 25-4801-82;
8) anti-mouse CD80, 1:300, eBioscience, APC, Clone 16-10A1, Cat 17-0801-82;
9) anti-mouse CD206, 1:200, Biolegend, PE, Clone C068C2, Cat 141705;
10) anti-mouse CD11c, 1:500, eBioscience, FITC, Clone N418, Cat 11-0114-82;
11) anti-mouse CD80, 1:300, eBioscience, PE, Clone 16-10A1, Cat 12-0801-82;
12) anti-mouse CD86, 1:300, eBioscience, APC, Clone GL1, Cat 17-0862-82;
13) anti-mouse MHC-II, 1:300, eBioscience, PE-Cyanine7, Clone M5/114.15.2, Cat 25-5321-82;
14) anti-mouse CD25, 1:200, eBioscience, APC, Clone PC61.5, Cat 17-0251-82;
15) anti-mouse Foxp3, 1:100, eBioscience, PE, Clone FJK-16s, Cat 12-5773-82;
16) anti-mouse CD44, 1:500, eBioscience, PE, Clone IM7, Cat 12-0441-82;
17) anti-mouse CD62L, 1:200, eBioscience, APC, Clone MEL-14, Cat 17-0621-82;
18) anti-calreticulin, 1:100, Cell Signaling Technology, Alexa Fluor 488, Cat 62304;
19) rabbit IgG isotype control, 1:100, Cell Signaling Technology, Alexa Fluor 488, Cat 2975.

Validation

No customized antibodies were used. Validation data of the antibodies purchased from commercial vendors are available on the manufactures' website and datasheets.

1) anti-mouse PD-1 (aPD-1), Bioxcell, Clone RMP1-14, Cat BE0146
https://bxcell.com/product/invivomab-anti-m-pd-1/
2) anti-mouse CD8 (aCD8), Bioxcell, Clone 53-6.7, Cat BE0004-1
https://bxcell.com/product/m-cd8a/
3) anti-GAPDH, Proteintech, Cat HRP-60004
https://www.ptgcn.com/products/GAPDH-Antibody-HRP-60004.htm
4) anti-HIF-1α, Novus Biologicals, Cat NB-100-105
https://www.novusbio.com/products/hif-1-alpha-antibody-h1alpha67_nb100-105
5) anti-CD3ε, Cell Signaling Technology, Cat 99940
https://www.cellsignal.com/products/primary-antibodies/cd3e-d4v8l-rabbit-mab/99940
6) anti-CD8α, Cell Signaling Technology, Cat 98941
https://www.cellsignal.com/products/primary-antibodies/cd8a-d4w2z-xp-rabbit-mab-mouse-specific/98941
7) anti-Foxp3, Cell Signaling Technology, Cat 12653
https://www.cellsignal.com/products/primary-antibodies/foxp3-d6o8r-rabbit-mab/12653
8) anti-granzyme B, Cell Signaling Technology, Cat 44153
https://www.cellsignal.com/products/primary-antibodies/granzyme-b-e5v2l-rabbit-mab-mouse-specific/44153
9) anti-phospho-histone H2AX (Ser139/Tyr142), Cell Signaling Technology, Cat 5438
https://www.cellsignal.com/products/primary-antibodies/phospho-histone-h2a-x-ser139-tyr142-antibody/5438
10) anti-calreticulin, Cell Signaling Technology, Cat 12238
https://www.cellsignal.com/products/primary-antibodies/calreticulin-d3e6-xp-rabbit-mab/12238
11) anti-HMGB1, Abcam, Cat ab18256
https://www.abcam.com/hmgb1-antibody-ab18256.html
12) anti-mouse CD45, eBioscience, perCP-Cyanine5.5, Clone 30-F11, Cat 45-0451-82
https://www.thermofisher.cn/cn/zh/antibody/product/CD45-Antibody-clone-30-F11-Monoclonal/45-0451-82
13) anti-mouse CD3e, eBioscience, FITC, Clone 145-2C11, Cat 11-0031-82
https://www.thermofisher.cn/cn/zh/antibody/product/CD3e-Antibody-clone-145-2C11-Monoclonal/11-0031-82
14) anti-mouse CD4, eBioscience, eFluor 450, Clone RM4-5, Cat 48-0042-82
https://www.thermofisher.cn/cn/zh/antibody/product/CD4-Antibody-clone-RM4-5-Monoclonal/48-0042-82
15) anti-mouse CD8a, Biolegend, APC, Clone 53-6.7, Cat 100712
https://www.biolegend.com/en-us/products/apc-anti-mouse-cd8a-antibody-150
16) anti-mouse CD8a, Biolegend, Alexa Fluor 594, Clone 53-6.7, Cat 100758
https://www.biolegend.com/en-us/products/alexa-fluor-594-anti-mouse-cd8a-antibody-9608
17) anti-mouse CD11b, Biolegend, FITC, Clone M1/70, Cat 101206
https://www.biolegend.com/en-us/products/fitc-anti-mouse-human-cd11b-antibody-347
18) anti-mouse F4/80, eBioscience, PE-Cyanine7, Clone BM8, Cat 25-4801-82

https://www.thermofisher.cn/cn/zh/antibody/product/F4-80-Antibody-clone-BM8-Monoclonal/25-4801-82
19) anti-mouse CD80, eBioscience, APC, Clone 16-10A1, Cat 17-0801-82
https://www.thermofisher.cn/cn/zh/antibody/product/CD80-B7-1-Antibody-clone-16-10A1-Monoclonal/17-0801-82
20) anti-mouse CD206, Biolegend, PE, Clone C068C2, Cat 141705
https://www.biolegend.com/en-us/products/pe-anti-mouse-cd206-mmr-antibody-7424
21) anti-mouse CD11c, eBioscience, FITC, Clone N418, Cat 11-0114-82
https://www.thermofisher.cn/cn/zh/antibody/product/CD11c-Antibody-clone-N418-Monoclonal/11-0114-82
22) anti-mouse CD80, eBioscience, PE, Clone 16-10A1, Cat 12-0801-82
https://www.thermofisher.cn/cn/zh/antibody/product/CD80-B7-1-Antibody-clone-16-10A1-Monoclonal/12-0801-82
23) anti-mouse CD86, eBioscience, APC, Clone GL1, Cat 17-0862-82
https://www.thermofisher.cn/cn/zh/antibody/product/CD86-B7-2-Antibody-clone-GL1-Monoclonal/17-0862-82
24) anti-mouse MHC-II, eBioscience, PE-Cyanine7, Clone M5/114.15.2, Cat 25-5321-82
https://www.thermofisher.cn/cn/zh/antibody/product/MHC-Class-II-I-A-I-E-Antibody-clone-M5-114-15-2-Monoclonal/25-5321-82
25) anti-mouse CD25, eBioscience, APC, Clone PC61.5, Cat 17-0251-82
https://www.thermofisher.cn/cn/zh/antibody/product/CD25-Antibody-clone-PC61-5-Monoclonal/17-0251-82
26) anti-mouse Foxp3, eBioscience, PE, Clone FJK-16s, Cat 12-5773-82
https://www.thermofisher.cn/cn/zh/antibody/product/FOXP3-Antibody-clone-FJK-16s-Monoclonal/12-5773-82
27) anti-mouse CD44, eBioscience, PE, Clone IM7, Cat 12-0441-82
https://www.thermofisher.cn/cn/zh/antibody/product/CD44-Antibody-clone-IM7-Monoclonal/12-0441-82
28) anti-mouse CD62L, eBioscience, APC, Clone MEL-14, Cat 17-0621-82
https://www.thermofisher.cn/cn/zh/antibody/product/CD62L-L-Selectin-Antibody-clone-MEL-14-Monoclonal/17-0621-82
29) anti-calreticulin, Cell Signaling Technology, Alexa Fluor 488, Cat 62304
https://www.cellsignal.com/products/antibody-conjugates/calreticulin-d3e6-xp-rabbit-mab-alexa-fluor-488-conjugate/62304
30) rabbit IgG isotype control, Cell Signaling Technology, Alexa Fluor 488, Cat 2975
https://www.cellsignal.com/products/antibody-conjugates/rabbit-da1e-mab-igg-xp-isotype-control-alexa-fluor-488-conjugate/2975
31) HRP-conjugated secondary antibody (anti-mouse), Cell Signaling Technology, Cat 8125
https://www.cellsignal.com/products/secondary-antibodies/boost-ihc-detection-reagent-hrp-mouse/8125
32) HRP-conjugated secondary antibody (anti-rabbit), Cell Signaling Technology, Cat 8114
https://www.cellsignal.com/products/secondary-antibodies/boost-ihc-detection-reagent-hrp-rabbit/8114
33) Fluorescence conjugated secondary antibody (anti-rabbit, DyLight 488), Abbkine, Cat A23220
https://www.abbkine.com/product/dylight-488-goat-anti-rabbit-igg-a23220/
34) Fluorescence conjugated secondary antibody (anti-rabbit, DyLight 594), Abbkine, Cat A23430
https://www.abbkine.com/product/dylight-594-rabbit-anti-goat-igg-a23430/

# Eukaryotic cell lines

Policy information about cell lines

| | |
|---|---|
| Cell line source(s) | The murine breast cancer cell line 4T1 (Cat CRL-2539) and the murine melanoma cell line B16F10 (Cat CRL-6475) were purchased from the American Type Culture Collection (ATCC). Human immortalized oral epithelial cell (HIOEC) was obained from the Shanghai Ninth People's Hospital. 4T1-Luc cell line was obtained from Prof. Xian-Zheng Zhang at Wuhan University. The original commercial source of the 4T1-Luc cell line was ATCC. |
| Authentication | Each cell line used was morphologically confirmed according to the information provided by culture collections. |
| Mycoplasma contamination | All the cell lines presented in this study were tested for mycoplasma contamination and they were free of mycoplasma contamination. |
| Commonly misidentified lines (See ICLAC register) | No commonly misidentified cell lines were used. |

# Animals and other organisms

Policy information about studies involving animals; ARRIVE guidelines recommended for reporting animal research

| | |
|---|---|
| Laboratory animals | Female BALB/c and C57BL/6 mice (6–8 weeks) were purchased from Hubei Provincial Academy of Preventive Medicine. |
| Wild animals | The study did not involve wild animals. |
| Field-collected samples | The study did not involve samples collected from field. |
| Ethics oversight | Ethical approval of this study was obtained from the Animal Ethics Committee of the School and Stomatology of Wuhan University. (approval number: S07920070E). All animal experimental procedures were performed in accordance with the Regulations for the Administration of Affairs Concerning Experimental Animals approved by the State Council of the People's Republic of China. |

Note that full information on the approval of the study protocol must also be provided in the manuscript.

# Flow Cytometry

## Plots

Confirm that:

- [x] The axis labels state the marker and fluorochrome used (e.g. CD4-FITC).
- [x] The axis scales are clearly visible. Include numbers along axes only for bottom left plot of group (a 'group' is an analysis of identical markers).
- [x] All plots are contour plots with outliers or pseudocolor plots.
- [x] A numerical value for number of cells or percentage (with statistics) is provided.

## Methodology

| | |
|---|---|
| Sample preparation | For lymphocyte analysis, tumors and tumor-draining lymph nodes were collected from mice and made into cell suspensions by gentleMACS™ dissociator and digestive enzyme (Miltenyi Biotec) according to the manufacturer's instructions. Then, the samples were passed through 200-mesh nylon mesh filters to obtain single-cell suspensions. Live or dead cells were separated by Fixable Viability Dye (eBioscience, eFluor 780). For all samples, cells were first stained with antibodies against surface antigens. In some experiments, cells were subsequently fixed, permeablized and stained for intracellular antigens. |
| Instrument | CytoFLEX flow cytometer (Beckman), MoFlo XDP cell sorter (Beckman) |
| Software | CytExpert v. 2.3 software (Beckman), FlowJo v. 10 (TreeStar) |
| Cell population abundance | The purity of the post-sorted cells was more than 95% as verified by flow cytometry. |
| Gating strategy | The gating strategy was shown in Supplementary Fig. 3a and Supplementary Fig. 9. Generally, single cell gates based on SSC-H and SSC-A, and FSC-H and FSC-A were used to exclude non-singlets. A live/dead cell gate based on fixable viability dye was used to exclude dead cells. |

- [x] Tick this box to confirm that a figure exemplifying the gating strategy is provided in the Supplementary Information.
